# Production of Collagens and Protein Hydrolysates with Antimicrobial and Antioxidant Activity from Sheep Slaughter By-Products

**DOI:** 10.3390/antiox11061173

**Published:** 2022-06-14

**Authors:** Alessandra Roseline Vidal, Rogério Luis Cansian, Renius de Oliveira Mello, Ivo Mottin Demiate, Aniela Pinto Kempka, Rosa Cristina Prestes Dornelles, José Manuel Lorenzo Rodriguez, Paulo Cezar Bastianello Campagnol

**Affiliations:** 1 Department of Science and Food Technology, Federal University of Santa Maria, Avenida Roraima, 1000, Santa Maria 97105-900, Brazil; renius.mello@ufsm.br (R.d.O.M.); rosa.prestes.dornelles@ufsm.br (R.C.P.D.); paulo.campagnol@ufsm.br (P.C.B.C.); 2 Departament of Food Engineering, Integrated Regional University of Alto Uruguai and Missões, Avenida Sete de Setembro, 1621, Erechim 99709-298, Brazil; cansian@uricer.edu.br; 3 Department of Food Engineering, State University of Ponta Grossa, Avenida General Carlos Cavalcanti, 4748, Ponta Grossa 84030-000, Brazil; demiate@uepg.br; 4 Department of Food Engineering, State University of Santa Catarina, Baixada Pioneira, SC-160, Pinhalzinho 89870-000, Brazil; aniela.kempka@udesc.br; 5 Technological Center of Meat of Galicia, Technological Park of Galicia, San Cibrán das Viñas, Rúa Galicia N 4, 32900 Ourense, Spain; jmlorenzo@ceteca.net

**Keywords:** enzymatic hydrolysis, Alcalase^®^, differential exploratory calorimetry, antioxidant capacity, electrophoresis, bioactive peptides

## Abstract

This work aimed to produce collagens and hydrolysates with antimicrobial and antioxidant activity from sheep slaughter by-products. The by-products (sheep and lamb) were treated and extracted. The collagens were hydrolyzed with the enzyme Alcalase^®^. The spectra of collagens and hydrolysates were similar (amide bands I, II, III, A, B). The bands presented by the collagens (α_1_, α_2_, β) were characteristic of type I collagen. The hydrolysates showed molecular weight peptides equal to/lower than 15 kDa. Collagens had a denaturation temperature of 39.32 (lamb) and 36.38 °C (sheep), whereas the hydrolysates did not undergo thermal transition. Hydrolysates showed lower values of antioxidant activity (AA) than the collagens. The collagens from lamb and from sheep displayed an AA of 13.4% (concentration of 0.0002%) and 13.1% (concentration of 0.0005%), respectively. At the concentration of 0.0020%, the lamb hydrolysates displayed an AA of 10.2%, whereas the sheep hydrolysates had an AA of only 1.98%. Collagen also showed higher antimicrobial activity compared to hydrolysates, requiring a lower concentration to inhibit the microorganisms tested. Sheep slaughter by-products proved to be a viable source for obtaining protein hydrolysates and collagens with antimicrobial and antioxidant activity, which can be applied in the development of nutraceuticals beneficial to human health.

## 1. Introduction

By-products consist of about 60% to 70% of slaughtered carcasses, and 20% are not edible [1]. Slaughtering by-products are generally used in the manufacture of fertilizers, fuels, and animal feed. However, the authors of recent studies have proven that these by-products can serve as raw materials for obtaining collagen and protein hydrolysates [2,3,4] since this protein prevails in the connective tissue of multicellular organisms [5].

The extraction of collagen and its derivatives from slaughter by-products has gained space since these proteins can be used in industrial processes to replace synthetic agents [6,7,8]. By enzymatic hydrolysis, using plant or animal proteases, protein hydrolysates can be obtained from by-products. Enzymatic hydrolysis can modify several functional groups and is used to increase the functionality of proteins through structural modification [9,10,11,12,13,14,15]. One of the enzymes most commonly used in the process of collagen hydrolysis is Alcalase^®^, which can be used at high temperatures, moderate alkalinity [16], and enable a cleaner hydrolysis process.

Research aimed at obtaining, characterizing, and fractionating collagen peptides and protein hydrolysates, has sought to analyze the functional properties of these compounds. According to some studies, these compounds can present antimicrobial, antioxidant, and antihypertensive activity [17,18,19,20,21,22]. The antimicrobial and antioxidant activity of collagens and protein hydrolysates is directly related to their structure and molecular composition. Peptide size and amino acid sequence directly influence the ability to chelate oxidizable metals.

In studies carried out by Zhang et al. [13] and Luo et al. [15], hydrolysates obtained from tuna heads (*Katsuwonus pelamis*) and collagen peptides from fish bones, respectively, were identified and evaluated for antioxidant activity. In the study by López-Pedrouso et al. [23], the antioxidant activity of porcine liver hydrolysates was evaluated, while in the study by Zheng et al. [24], bovine bone hydrolysates were evaluated. It appears that several studies seek to evaluate the antioxidant activity of hydrolysates from different sources, such as fish, bovine, porcine, and poultry.

However, there is a gap in the literature regarding the evaluation of the antioxidant and antimicrobial activity of collagens and hydrolysates obtained from sheep slaughter by-products (cartilage, carcass and meat trimming, and bone scraps), which are not used by the industry, being discarded or sold (low market value) for feed production. These collagens and hydrolysates can be used as substitutes for those found commercially since sheep meat does not have Bovine Spongiform Encephalopathy (BSE), is consumed in Muslim countries, and being of mammalian origin, is expected to present greater thermal stability in relation to fish protein.

Taking this into account, the objective of the study was to produce collagens and hydrolysates from sheep slaughter by-products and evaluate their antioxidant and antimicrobial activity for the first time, allowing the insertion of new products into the market, improving diet, and contributing to human health, besides valuing and enabling a suitable destination for this by-product.

## 2. Materials and Methods

The Department of Animal Science of the Federal University of Santa Maria and the company *Chibito–Rancho Taura Alimentos* have donated the by-products of sheep slaughter (RAO) (bones scrapings, cartilages, carcass and meat trimming). Two groups of by-product samples were used, as follows: sheep slaughter by-product (SS) (*Merino*, *Texel*, *Ideal*, *Corriedale*, and other breeds; weight from 45 to 60 kg and aged 1 to 2 years) and lamb slaughter by-product (SL) (*Texel x Ile de France* breed; weight from 28 to 34 kg and aged 90 days to 6 months).

Raw materials were prepared and stored according to Vidal et al. [25]. Scrapings, cartilages, and trimmings of carcasses and cuts were ground in a meat grinder using 10 and 5 mm discs and were stored at −22 °C. The Alcalase 2.4 L^®^ enzyme (company *Tovanni Benzaquen Ingredientes*, Novozymes^®^, São Paulo, Brazil) was used to obtain the hydrolysates. Enzymes, all reagents, and standards are commercially available and are of analytical grade (AG).

### 2.1. Extraction of Collagens

#### 2.1.1. Pretreatment of Samples

The methodologies described by Li et al. [26] and Duan et al. [27], with modifications according to Vidal et al. [25], were used for the treatment of by-products. The samples were treated with 0.1M sodium hydroxide (NaOH) solution (2 days), 0.5M disodium ethylenediaminetetraacetic acid (EDTA-2N) solution (5 days), and 10% butyl alcohol solution (2 days). All steps were performed under stirring (150 rpm and 4 °C) in a Shaker incubator (Solab SL-223, Piracicaba, Brazil). After pretreatment, samples were referred to extraction.

#### 2.1.2. Enzyme Extraction

The methodology described by Kittiphattanabawon et al. [28], with modifications according to Vidal et al. [25], was used to extract pepsin-soluble collagen. Extraction was performed with0.5M acetic acid solution (1:15 (*w*/*v*)), containing the pepsin enzyme (1:2.50 enzyme/protein ratio; powder, ≥400 units/mg protein, Sigma, St. Louis, MO, USA), was used for collagen extraction. The mixture was continuously stirred (150 rpm and 4 °C) in a Shaker incubator (SL-223, Solab, Piracicaba, Brazil) for 3 days. Frozen collagens (–22 °C, VF50F, Metalfrio) were freeze-dried (LS 3000, Terroni, São Carlos, Brazil).

### 2.2. Obtaining Protein Hydrolysates

The methodology described by Schmidt and Salas-Mellado [29] with modifications, using the Alcalase^®^ enzyme, was used for the enzymatic reactions. Hydrolysis reactions were performed in closed glass vials, immersed in an ultra thermostatic bath (2000 W power, Model SL152, SOLAB, Piracicaba, Brazil). Samples (4% (*m*/*v*) of collagen in relation to the buffer volume) were dissolved in a phosphate buffer, pH-value 7.5, and 8% (v/m) of the enzyme in relation to the collagen mass were added. The hydrolysis reaction (55 °C) occurred for 2 h, being inactivated (85 °C) for 15 min. Finally, the frozen hydrolysates (–22 °C, VF50F, Metalfrio) were freeze-dried (LS 3000, Terroni, São Carlos, Brazil).

### 2.3. Protein Content of Collagen Hydrolysates

To determine the protein content of hydrolysates, the methods of Lowry and modified biuret were used, as described by Lowry et al. [30] and Di Bernardini et al. [31], respectively. As a standard for the methods, bovine albumin manufactured by Sigma-Aldrich (São Paulo, Brazil) was used.

### 2.4. Fourier Transform Infrared Spectroscopy (FTIR) Analysis

FTIR analysis was used to structurally characterize the collagens and hydrolysates. Following the methodology described by Demiate et al. [32], 16 scans were established per spectrum (2 cm^−1^ resolution), in attenuated total reflectance (ATR) (400 to 4000 cm^−1^), using Shimadzu IR Prestige-21 equipment (Shimadzu Corporation, Kyoto, Japan).

### 2.5. Electrophoresis (SDS-PAGE) Analysis

The methodology described by Laemmli [33], with modifications according to Vidal et al. [25], was used for SDS-PAGE analysis. Distilled water was used to prepare the sample solutions. Electrophoresis analysis (150 V and 30 mA, for 2h, vertical vat) was performed with stacking gel (5%), running gel (15%), protein standard (15 μL), and sample (20 μL).

### 2.6. Denaturation Temperature

Differential scanning calorimetry (DSC) curves were determined according to Schmidt et al. [2], using DSC-Q200 equipment (TA-Instruments, New Castle, DE, USA). The heating range used was −20 to 100 °C and the heating rate was 10 °C min^−1^.

### 2.7. Antioxidant Activity (DPPH)

The analysis of DPPH (2,2-diphenyl−1-picrylhydrazyl), as described by Brand-Willians et al. [34] and modified by Sánchez-Moreno et al. [35], was used to evaluate the antioxidant activity of collagens and hydrolysates. The UV spectrophotometer, model UV–M51 (SERVYLAB, São Leopoldo, Brazil) was used in the analysis. The sample concentrations tested were from 0.0001% to 0.25% (the samples were diluted in distilled water). To perform the analysis of antioxidant activity, an ethanolic solution (80% *v*/*v*) of DPPH (78.86 µg/mL (*m*/*v*)) was used. In test tubes containing 2 mL of ethanolic DPPH solution, 2 mL of each sample solution were added, and then they were homogenized and left to rest for 30 min at room temperature and protected from light. For the blank, 4 mL of distilled water was used and for the control sample, a solution containing 2 mL of distilled water and 2 mL of ethanolic DPPH solution was used. Then, the samples were read in a spectrophotometer (previously tared with the blank) at 517 nm. The DPPH radical scavenging activity (AA) was calculated using the following equation:

$$AA\;\left(\%\right)=\left(\frac{Control\;Absorbance-Sample\;Absorbance}{Control\;Absorbance}\right)\times 100$$

### 2.8. Antimicrobial Activity (MIC)

The antimicrobial activity of the samples was determined through the analysis of minimum inhibitory concentration (MIC), using the indirect method of bacterial growth in a liquid culture medium [36]. MIC determination was performed on Gram-negative (*Escherichia coli*–ATCC 25922; *Salmonella choleraesuis*–ATTC 10708; *Klebsiella pneumoniae*–ATCC 10031; *Enterobacter cloacae*–ATCC 13047; *Pseudomonas aeruginosa*–ATCC 27853) and Gram-positive (*Enterococcus faecalis*–ATCC 29212; *Staphylococcus aureus*–ATCC 25923; *Bacillus subtilis*–ATCC 6633; *Listeria monocytogenes*–ATCC 7644; *Streptococcus mutans*–ATCC 25175) bacteria. The MIC was determined as the lowest percentage of collagen hydrolysate in relation to the volume of broth that did not generate changes in turbidity, obtained from the difference between the readings (490 nm) performed at 24 and 0 h.

### 2.9. Statistical Evaluation

The collagen extractions were repeated at least three times and the analyses were performed in triplicate. Univariate analysis of variance (ANOVA) and the *t*-test or Tukey’s test (5% significance level, Statistica^®^ 8.0 software-StatSoft Inc., Tulsa, OK, USA) were used for data comparison and analysis. FTIR spectra were treated according to Vidal et al. [25] and analyzed statistically with the SAS^®^220 System for Windows ™ version 9.4 (SAS Institute Inc., Cary, NC, USA).

## 3. Results

### 3.1. Protein Content of Hydrolysates

Protein content did not differ (*p* > 0.05) between the samples according to the Lowry analysis; on the other hand, by the biuret analysis (*p* < 0.05), it did—the protein content for the sheep hydrolysate sample was higher than that of the lamb hydrolysate (Table 1). In other studies, different bovine collagens (collagen fiber, fiber powder, gelatins, and hydrolysates) were hydrolyzed under distinct conditions using different enzymes, Alcalase (*Tovanni Benzaquen Ingredientes*, Novozymes^®^, São Paulo, Brazil), Flavourzyme (*Tovanni Benzaquen Ingredientes*, Novozymes^®^, São Paulo, Brazil), and pepsin (Sigma, St. Louis, MO, USA) [12,18,37]. Protein content obtained for the samples hydrolyzed with Alcalase (biuret) ranged from 7.06 to 10.2 mg/mL; with Flavourzyme (Lowry), from 3.89 to 8.64 mg/mL; and with pepsin (Lowry), from 4.51 to 8.70 mg/mL, values lower than those found in our study.

The authors reported that differences between protein contents were related to the enzyme, which can act in different ways depending on the conditions imposed on it such as pH-value, temperature, enzyme/substrate ratio, type of substrate, and the form of ultrasound used in the hydrolysis process. Collagen hydrolysates displayed good protein content, allowing their use in various industrial sectors (food, pharmaceutical, cosmetic).

### 3.2. Fourier Transform Infrared Spectroscopy (FTIR) Analysis

**Figure 1:**
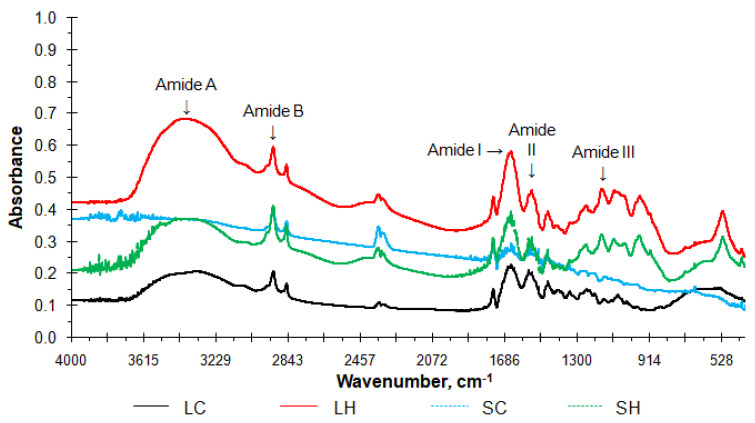
FTIR spectra of collagens and hydrolysates from sheep slaughter by−products (LC−lamb collagen; SC−sheep collagen; LH−lamb hydrolysate; SH−sheep hydrolysate).

The major peaks of the infrared spectra of the collagens and hydrolysates are shown in Figure 1. LC and SC were similar, as were LH and SH, the samples showed spectra with bands at similar frequencies. With peaks located in the regions of amide I, II and III, A and B and bands, the collagens and hydrolysates showed an FTIR spectra similar to those found by Ata et al. [4] for sheep feet collagen and by Schmidt et al. [2] for collagen and hydrolysates obtained from mechanically separated meat waste. The amide A bands (NH free stretching vibrations of a hydrogen bond [38,39]) were found at wave numbers of 3312, 3314, 3386, and 3401 cm^−1^ for SC, LC, SH, and LH, respectively. The amide B bands of SC (2920 cm^−1^), LC (2916 cm^−1^), SH (2920 cm^−1^), and LH (2924 cm^−1^), on the other hand, are related to the absorption due to the alkyl CH_2_ chain and the asymmetric stretching vibration of CH_2_ [40,41].

The amide I bands, that contribute to the secondary structure of the peptide (associated with the stretching vibrations of the carbonyl groups) [42], were found at wave numbers 1636 (SC), 1630 (LC), 1647 (SH), and 1647 (LH) cm^−1^. The peaks at 1541 and 1250 cm^−1^ for SC, at 1539 and 1238 cm^−1^ for LC, at 1522 and 1155 cm^−1^ for SH, and at 1539 and 1155 cm^−1^ for LH, are associated with the triple helix structure of collagen (N-H bending vibrations coupled with C-N stretching vibration) [41,43]. The FTIR spectra of the hydrolyzed collagens showed medium intensity peaks at wave numbers lower than 1200 cm^−1^, differing from the spectra of the raw collagens. Thus, this outcome indicates that the hydrolysis process modified the molecular structure of collagens, interfering with their functionality, however, it did not negatively affect the structure of the product, maintaining the necessary links for its good quality.

### 3.3. Electrophoresis Analysis (SDS-PAGE)

**Figure 2:**
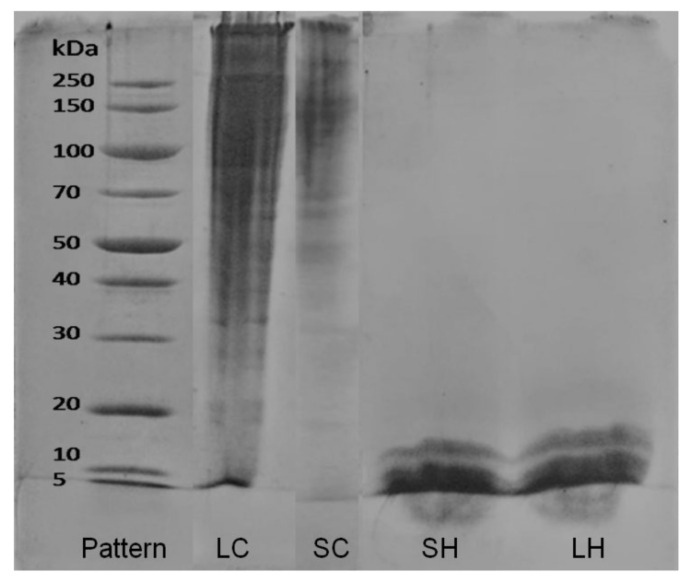
Electrophoresis (SDS-PAGE) of sheep (SC) and lamb (LC) collagens; and of sheep (SH) and lamb (LH) hydrolysates.

In the analysis of electrophoresis, proteins are separated according to their masses or loads (Figure 2). The SDS-PAGE of collagens presented distinct bands in the range from >250 to 5 kDa, presenting α_1_, α_2_, and β chains (two α-linked chains), which is expected for type I collagen [4,25,44]. This molecular weight difference is related to the use of the pepsin enzyme in the collagen extraction process, which hydrolyzes peptide bonds.

SDS-PAGE of hydrolysates proves that the Alcalase enzyme has hydrolyzed the collagens, generating peptides with lower molecular weight, with a mass equal to/lower than 15 kDa. Similar results were found by Schmidt et al. [2] for collagens from mechanically separated meat residues hydrolyzed with the enzymes Flavourzyme (*Tovanni Benzaquen Ingredientes*, Novozymes^®^, São Paulo, Brazil) and Alcalase (*Tovanni Benzaquen Ingredientes*, Novozymes^®^, São Paulo, Brazil). The hydrolysates showed clear bands below 15 KDa, which confirmed the hydrolysis of the collagen molecule into peptides.

The authors concluded that the use of more severe hydrolysis conditions, such as temperatures above denaturation, broke the triple helix structure of collagen and peptide bonds, generating lower molecular weights. In addition, differences in molecular weight and the number of generated peptides are directly influenced by the enzyme applied in the hydrolysis of the sample: the greater the activity of the enzyme, the greater the breakdown of proteins in smaller peptides [37]. The hydrolysates were not purified, as the hydrolysis was sufficient to generate peptides of low molecular weight and standardized (≤15 kDa), as shown by electrophoresis analysis.

### 3.4. Denaturation Temperature

**Figure 3:**
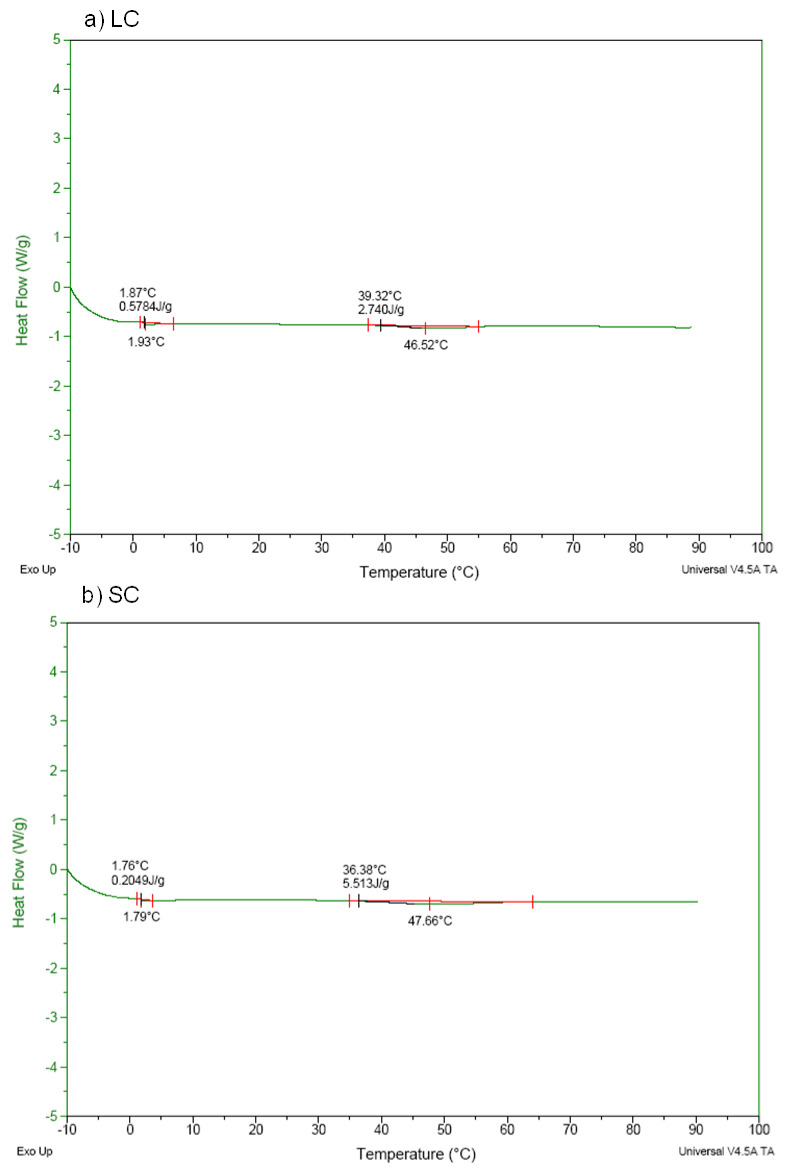
Differential scanning calorimetry (DSC) thermogram of the (**a**) lamb (LC) and (**b**) sheep (SC) collagen samples.

**Figure 4:**
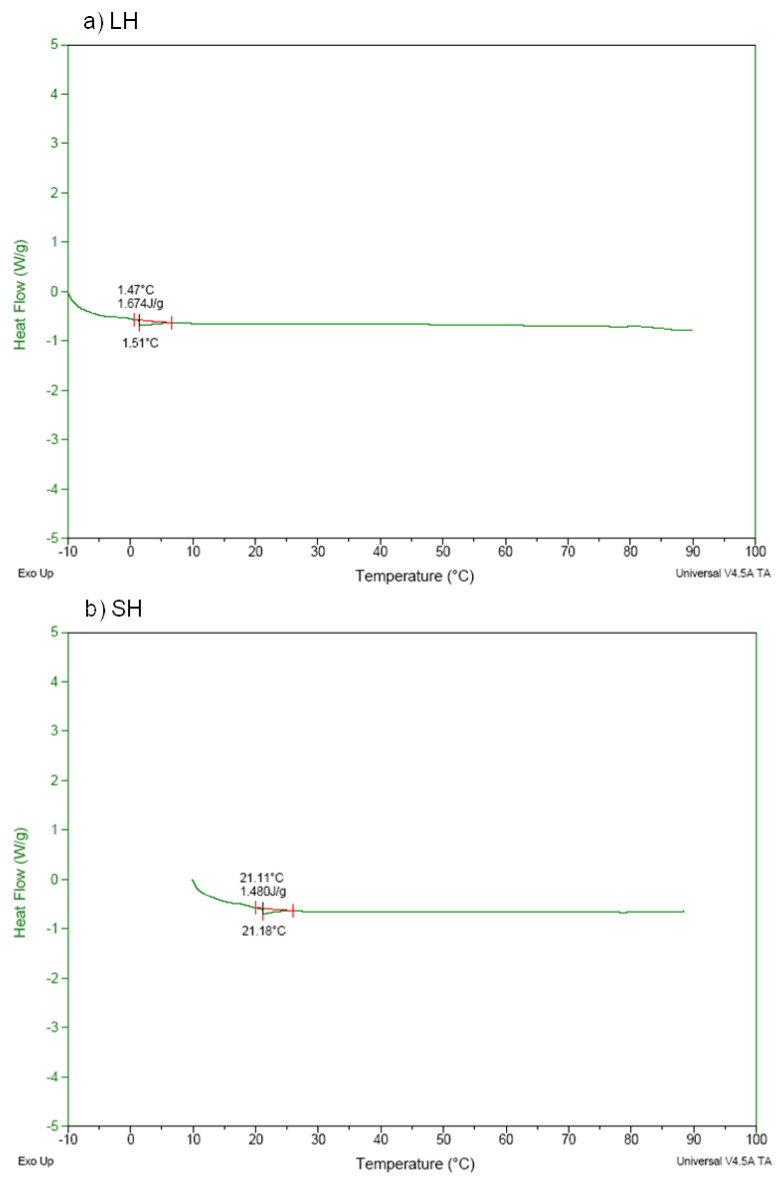
Differential scanning calorimetry (DSC) thermogram of the (**a**) lamb (LH) and (**b**) sheep (SH) hydrolysates.

Figure 3 and Figure 4 show the DSC thermograms for the collagens and the hydrolysates obtained from sheep slaughter by-products. In the thermograms of the crude collagens samples, lamb (Figure 3a) and sheep (Figure 3b), the endothermic transition was observed at 39.32 °C and 36.38 °C, respectively, similar to the results obtained for denaturation of collagens-II from chicken sternal cartilage (44.83 to 53.51 °C) [3] and collagens from snakehead (*Channaargus*) skin (34.1 and 34.2 °C) [45].

The thermograms of the hydrolyzed collagens samples (Figure 4a,b), on the other hand, showed no thermal transition phenomena, showing that the collagen molecules were hydrolyzed into smaller peptides during hydrolysis, which was caused by the action of the heat and enzyme. This outcome is similar to the results obtained for hydrolyzed collagens samples from mechanically separated meat waste [2].

The denaturation temperature values found for the collagens allow their use in the development of various food products, aimed at improving technological, sensory, functional, and nutritional properties. Hydrolysates, due to their denaturing process, lose some technological properties, such as gel formation, but can be used for nutritional and functional improvement of foods.

### 3.5. Antioxidant Activity (DPPH)

The lamb collagen showed a higher (*p* < 0.05) antioxidant activity value, similar to that of the sheep collagen, although in different dilutions, 0.0002 and 0.0005%, respectively (Table 2). Higher antioxidant activity was found by Vidal et al. [37] for different hydrolysates of bovine collagens (fiber, fiber powder, gelatins, and hydrolysates) with the pepsin enzyme, ranging from 14.6 to 53.7%. In another study conducted by Chen et al. [41] for collagen samples extracted from *Nibea japonica* swim bladders, the antioxidant activity ranged from 0 to 50%.

Collagen showed higher (*p* < 0.05) values of antioxidant activity than hydrolysates, probably because they already had short-chain peptides with good functionality, a fact confirmed by the electrophoresis analysis. In contrast, the sample of horse mackerel hydrolysate, evaluated by García-Moreno et al. [46], showed elimination activity of the DPPH radical between 35 and 45%, depending on the type of treatment employed.

The sheep hydrolysate was the sample that presented the lowest (*p* > 0.05) antioxidant activity value. Probably, the excessive hydrolysis of the collagen protein structure generated peptides with low functionality. The exaggerated or uncontrolled breakdown of proteins may impair the functionality of hydrolysates by generating non-functional or poorly-functional peptides, or even inactive or free amino acids [46]. In addition, the release of peptides with antioxidant capacity is related to the choice of enzymatic treatment, properties of the substrate, conditions of the process (temperature, pH-value, and enzyme/substrate ratio), and mainly the extension of the hydrolysis reaction [14,37].

In the study by He et al. [19], gelatin hydrolysates were obtained using ultrasound in the hydrolysis process at different frequencies (200, 300, and 400 W). The hydrolysates were evaluated for DPPH radical scavenging capacity. It was observed that the control hydrolysate (without ultrasound) showed antioxidant activity of around 28%, whereas that obtained with ultrasound at a frequency of 300 W, was around 45% and the hydrolysate with ultrasound at a frequency of 400 W had activity reduced to around 30%.

The collagens, according to the electrophoresis analysis (3.3), showed a variation in molecular weight, generating peptides of various sizes. This fact may have benefited the antioxidant activity and generated peptides with greater functionality. The hydrolysates had a molecular weight equal to or less than 15 kDa and lower antioxidant activity than the collagens. Some studies have reported that the purification of hydrolysates improves the antioxidant activity [15,47,48,49]; as it fractionates the peptides into lower molecular weights, which have greater functionality.

### 3.6. Antimicrobial Activity (MIC)

All samples of collagens and hydrolysates showed antimicrobial activity regarding the tested bacteria (Table 3).

Collagens were more effective in inhibiting bacteria when compared with hydrolysates, requiring a smaller amount of sample for inhibition. Hydrolysates showed higher antimicrobial activity when compared with hydrolysates of bovine collagens studied by Vidal et al. [18], using the Alcalase enzyme in the hydrolysis process. Samples were tested with *Salmonella choleraesuis* and *Staphylococcus aureus* bacteria, requiring concentrations of 20% and 17.5%, respectively, for inhibiting the microorganisms. In another study conducted by Vidal et al. [12] using the Flavourzyme enzyme in the hydrolysis process of these same samples and microorganisms for testing, MIC values ranged from <10 to >25%.

In the study by Rashid et al. [50], fish protein hydrolysates showed inhibitory capacity against *Salmonella typhimurium*, *Listeria innocua*, *Escherichia coli*, and *Listeria monocytogenes*. The highest antimicrobial activity of the studied samples may have occurred due to the size of the generated peptides. According to Kim and Wijesekara [51], small peptides (molecular weight less than 10 kDa), with a positive load and composed of amphipathic molecules (hydrophilic and hydrophobic regions) have antimicrobial activity [52]. Moreover, the peptide permeation depends on the amino acid sequence, the composition of the lipid membrane, and the peptide concentration [53].

The tested samples showed greater antimicrobial activity when compared to collagens and hydrolysates from other sources, showing an excellent application potential for inhibiting the tested microorganisms.

## 4. Conclusions

The production of collagens and hydrolysates with antimicrobial and antioxidant activity from sheep slaughter by-products was possible. Samples of hydrolysates presented similar protein contents. Collagens and hydrolysates showed spectra with peaks located in the regions of the amide bands I, II, III, A, and B. The collagens presented bands characteristic of collagen type I, α_1_, α_2_, and β. The hydrolysates showed peptides of low molecular weight equal to/lower than 15 kDa. The collagen’s denaturation temperature was 39.32 °C (lamb) and 36.38 °C (sheep). The hydrolysates did not undergo thermal transition.

The highest antioxidant activity found for lamb and sheep collagen was 13.4% (0.0002%) and 13.1% (0.0005%), respectively; for lamb and sheep hydrolysates it was10.2% and 1.98% (both at 0.0020%), respectively. All samples of collagens and hydrolysates showed antimicrobial activity concerning the tested bacteria.

Collagen and hydrolysates showed good antimicrobial and antioxidant activity. The sample concentrations used for antioxidant evaluation were low when compared to other similar works, and the concentrations necessary to inhibit the growth of the tested bacteria were low compared to products (collagen and hydrolysates) from other sources.

In this way, collagens and hydrolysates obtained from sheep slaughter by-products stand out in relation to others already studied, because, in addition to contributing to environmental sustainability (appropriate destination of a by-product), they make it possible to obtain value-added products, which present, in addition to properties applicable to the development and improvement of new products and food ingredients, the possibility of use in human health. However, because they are recently studied products, they need more research focused on their use and application.

## Figures and Tables

**Table 1:** Mean protein content found for hydrolysates of lamb and sheep collagen.

Hydrolysates	Lowry (mg/mL)	Biuret (mg/mL)
Lamb	17.4 ^aA^ ± 2.51	11.7 ^bB^ ± 0.06
Sheep	17.4 ^aA^ ± 0.80	12.2 ^aB^ ± 0.23

Means ± standard deviation. Means followed by distinct lowercase letters in the same column differ (*p* < 0.05) from each other according to the *t*-test. Means followed by distinct uppercase letters in the same line differ (*p* < 0.05) from each other according to the *t*-test.

**Table 2:** Antioxidant activity (DPPH, %) of collagens and hydrolysates obtained from sheep slaughter by-products.

Concentration (%)	DPPH (%)			
	Collagen		Hydrolysate	
	Lamb	Sheep	Lamb	Sheep
0.2500	0.00 ^gB^ ± 0.00	0.00 ^gB^ ± 0.00	4.65 ^bA^ ± 0.59	0.00 ^gB^ ± 0.00
0.1250	0.00 ^gA^ ± 0.00	0.00 ^gA^ ± 0.00	0.00 ^eA^ ± 0.00	0.00 ^gA^ ± 0.00
0.0625	0.00 ^gC^ ± 0.00	4.28 ^fA^ ± 0.27	0.00 ^eC^ ± 0.00	0.76 ^fB^ ± 0.10
0.0313	5.07 ^dA^ ± 0.10	4.10 ^fB^ ± 0.11	0.00 ^eD^ ± 0.00	1.27 ^deC^ ± 0.09
0.0156	3.60 ^eB^ ± 0.32	8.64 ^cA^ ± 0.29	0.11 ^eD^ ± 0.08	1.47 ^cdC^ ± 0.06
0.0078	1.63 ^fC^ ± 0.27	7.89 ^dA^ ± 0.10	2.58 ^cB^ ± 0.29	0.00 ^gD^ ± 0.00
0.0039	1.56 ^fB^ ± 0.04	8.23 ^cdA^ ± 0.13	0.99 ^dC^ ± 0.15	1.64 ^bcB^ ± 0.13
0.0020	12.5 ^bA^ ± 0.16	11.8 ^bA^ ± 0.39	10.2 ^aB^ ± 0.37	1.98 ^aC^ ± 0.13
0.0010	12.1 ^bA^ ± 0.15	8.25 ^cdB^ ± 0.22	0.00 ^eD^ ± 0.00	1.61 ^bcC^ ± 0.08
0.0005	10.7 ^cB^ ± 0.13	13.1 ^aA^ ± 0.12	0.55 ^deD^ ± 0.06	1.75 ^abcC^ ± 0.08
0.0002	13.4 ^aA^ ± 0.41	6.60 ^eB^ ± 0.27	2.69 ^cC^ ± 0.27	1.04 ^efD^ ± 0.18
0.0001	0.00 ^gC^ ± 0.00	7.10 ^eA^ ± 0.19	2.03 ^cB^ ± 0.09	1.85 ^abB^ ± 0.14

Means ± standard deviation. Means followed by distinct lowercase letters in the same column differ (*p* < 0.05) from each other according to Tukey’s test. Means followed by distinct uppercase letters in the same line differ (*p* < 0.05) from each other according to Tukey’s test.

**Table 3:** Minimum inhibitory concentration (%) of samples of collagens and hydrolysates to inhibit the growth of Gram-positive and Gram-negative bacteria.

Bacteria	Collagen		Hydrolysate	
Gram-Positive	Lamb	Sheep	Lamb	Sheep
*Listeria monocytogenes*	5.25	1.31	7.50	7.50
*Staphylococcus aureus*	2.63	1.31	6.00	6.00
*Bacillus subtilis*	2.63	2.63	5.25	3.75
*Streptococcus mutans*	0.66	2.63	6.00	5.25
*Enterococcus faecalis*	2.63	1.31	6.00	6.00
**Gram-Negative**				
*Escherichia coli*	2.63	2.63	6.00	6.00
*Salmonella choleraesuis*	2.63	1.31	9.00	6.00
*Klebsiella pneumoniae*	1.31	2.63	6.00	5.25
*Pseudomonas aeruginosa*	0.66	0.66	3.00	1.88
*Enterobacter cloacae*	2.63	2.63	6.00	10.5

## Data Availability

Data is contained within the article.
